# Rare invasive inflammatory fibroid polyp presenting as small bowel intussusception: Two case reports and review of the literature

**DOI:** 10.1097/MD.0000000000041956

**Published:** 2025-03-28

**Authors:** Yuqiang Tang, Xinye Cui, Zhengdong Zhao, Yu Chen, Boyang Dai, Fuwen Luo

**Affiliations:** a Department of Acute Abdominal Surgery, The Second Hospital of Dalian Medical University, Dalian, Liaoning Province, People’s Republic of China; b Department of General Surgery, Beijing Friendship Hospital, Capital Medical University, Beijing, People’s Republic of China.

**Keywords:** bowel intussusception, immunohistochemistry, pathology, Vanek tumor.

## Abstract

**Rationale:**

Inflammatory fibroid polyp (IFP) is a rare benign neoplasm of uncertain etiology and mostly occurs in the stomach, emerging from the submucosal layers. Intussusception causing bowel obstruction due to IFP is even rare. We present 2 cases of IFP in adults, which caused small bowel intussusception and broke through the submucosa uncommonly.

**Patient concerns:**

This article reports 2 patients presenting with abdominal pain. One patient was an 81-year-old Chinese man, who presented with a 7-day history of intermittent left abdominal pain. The other patient was a 49-year-old Chinese woman with a 5-day history of intermittent lower abdominal distension pain. Their abdominal computed tomography both demonstrated small bowel intussusception.

**Diagnoses:**

The 2 patients were diagnosed with small bowel intussusception.

**Interventions:**

Both patients underwent surgical resection of a segment of the small intestine. During the old man’s operation, a 2.5 cm × 3.5 cm polypoid tumor was found in the jejunum, at a distance of 60 cm from the ligament of Treitz. During the women’s operation, a 3.6 × 3.7 cm polypoid lesion was found in the ileum, which protruded into, and completely occluded the lumen.

**Outcomes:**

The 2 patients had an uneventful recovery, being discharged about 1 week postoperatively without any postoperative complications. Morphologically, the old man’s intraluminal intestinal mass had invaded muscularis propria, and was negative for CD34 immunohistochemically, creating difficulties in diagnosing IFP. The woman’s intraluminal intestinal mass had infiltrated into the serosal layer. Ultimately, the pathological diagnosis for both patients was IFP.

**Lessons:**

We described 2 rare cases of small bowel intussusception caused by IFP. IFP commonly involves only the submucosa, rarely breaks through the submucosa, and invades the muscularis propria and subserosa layer. Its invasive nature is extremely rare and may provide additional evidence to support the neoplastic nature of IFP. Besides, a differential diagnosis is essential When an IFP is negative for CD34 expression.

## 1. Introduction

Inflammatory fibroid polyp (IFP) is a rare benign polypoid lesion of the digestive system originating from mesenchymal tissue, which was first reported by Vanek in 1949 as “submucosal granulomas with eosinophilic infiltrate”.^[1]^ It is usually solitary, comes from epithelial tissue, and is always located in the gastric antrum and small bowel.^[2]^ Smaller lesions of IFP are generally asymptomatic, and often detected during gastroscopy and colonoscopy. When IFP grows to a certain size, it can cause gastrointestinal obstruction, even intussusception, presenting with gastrointestinal symptoms including abdominal pain, distention, nausea, vomiting, cessation of exhaust, cessation of defecation, and even hematochezia.^[2,3]^ Intussusception is telescoping a segment of the gastrointestinal tract into the lumen of an adjacent segment. Intussusception occurs infrequently in adults, with an incidence of 2 to 3 cases per 1,000,000.^[4]^ Adult intussusception is mostly secondary, due to lesions in the intestinal lumen or on the intestinal wall causing dysrhythmia of peristalsis, and strong peristalsis of the proximal bowel sends the lesion together with the bowel into the distal bowel, and common pathological factors include tumor, polyp, diverticulum, inflammation, adhesion, and foreign body in the intestinal lumen.^[5,6]^ Its diagnosis is difficult due to nonspecific signs and symptoms, so early diagnosis and treatment are vital for the prognosis. IFP commonly involves only the submucosa, rarely breaks through the submucosa, and invades the muscularis propria and subserosa layer. Here we report 2 rare cases of adult small bowel intussusception caused by IFP, which breaks through the submucosa uncommonly. We have also performed a literature review of these rare conditions.

## 2. Case reports

### 2.1. Case 1

**Figure 1.**
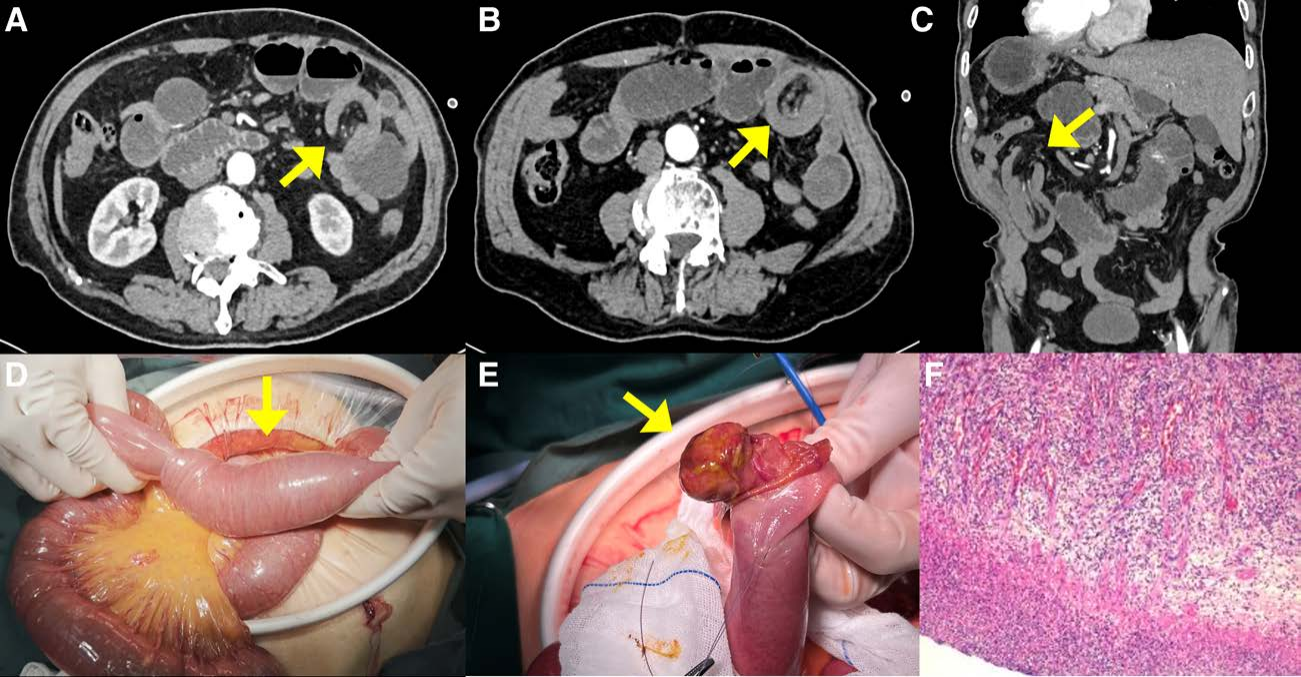
(A–F) Radiographic features in the perioperative period, intraoperative exhibition and postoperative histopathological features of case 1. (A, B, C) Abdominal CT showed the “target sign,” and “cuppy sign” of intestinal intussusception (yellow arrow). (D) Surgical exploration revealed the proximal portion of the bowel and its mesentery into a more distal segment. (E) A 3.5 cm × 2.5 cm lesion (yellow arrow) was observed. (F) Microscopic examination showed that the lesion invaded muscularis propria and a background of inflammatory cells with eosinophils was observed. CT = computed tomography.

An 81-year-old Chinese man presented with a 7-day history of intermittent left abdominal pain accompanied by nausea and vomiting. He had his last defecation and exhaust 2 days before the admission. Computed tomography (CT) of the abdomen demonstrated small bowel obstruction in the left upper quadrant abdomen, a segment of small intestine mesentery inserted into another intestine, high possibility of intussusception (Fig. 1A–C). The patient subsequently underwent a laparotomy in the operating room, and a segment jejunojejunal intussusception was identified, caused by a 2.5 cm × 3.5 cm polypoid tumor, at a distance of 60 cm from the ligament of Treitz (Fig. 1D and E). The patient underwent surgical resection of a segment of the small intestine, and the surgical procedure was completed in 2 hours, with an estimated blood loss of approximately 30 mL. The patient experienced a smooth recovery and was discharged 1 week after the surgery. The patient was closely followed up for 2 years postoperatively, including a follow-up abdominal CT scan, which revealed no evidence of recurrence.

Pathologic examination of the lesion showed the polypoid tumor invaded the muscularis propria. The intestinal mucosa was eroded, with granulation tissue formation underneath, and was richly vascularized, with a perivascular visible scattered distribution of bulky and heterocysts with pronounced nucleoli, visible nuclear division, and abundant cytoplasm (Fig. 1F). Immunohistochemistry revealed that the heterocyte was positive for ALK, Ki-67, and B-Catenin and negative for HMB45, CD117, CD34, SMA, Desmin, DOG1, CD31, and ERG. Combined with immunohistochemical staining, it was considered a junctional or low-grade mesenchymal tumor, and further consultation was recommended. The diagnosis was confirmed after consultation with the pathology department of Fudan University Affiliated Cancer Hospital. Immunohistochemistry showed the spindle cells were weakly positive for CD34, positive for Ki-67, and negative for STAT6, DOG-1, AE1/AE3, ALK (Ventana-D5F3), GFAP, S-100, SOX10. Proliferative mesenchymal cells were seen in the submucosa in the form of spindle and stellate with eosinophilic infiltration, consistent with IFP.

### 2.2. Case 2

**Figure 2.**
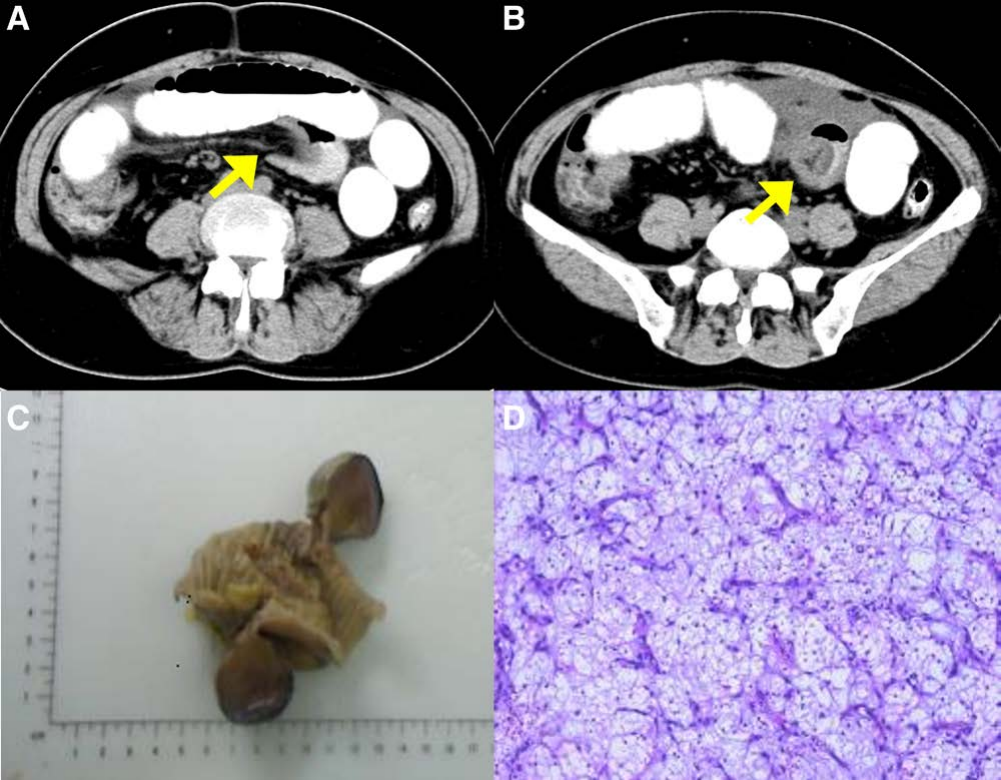
(A–D) Radiographic features in the perioperative period and postoperative histopathological features of case 2. (A and B) Abdominal CT showed the “target sign” and “cuppy sign” of intestinal intussusception (yellow arrow). (C) Gross specimen of tumor: a 3.6 × 3.7 cm polypoid lesion was observed, invading the serosa layer. (D) A background of a significant number of inflammatory cells with a predominance of lymphocytes, plasma cells, and eosinophils was seen. CT = computed tomography.

A 49-year-old Chinese woman presented with a 5-day history of intermittent lower abdominal distension pain, especially periumbilical abdomen. Abdominal CT showed a segment of the small intestine inserted into another in the left lower abdomen and a partial accumulation of fluid and gas in the intestines (Fig. 2A and B). The patient received an exploratory laparotomy exploration in an emergency. At laparotomy, an ileal intussusception was identified at a 40 cm distance from the ileocecum. After manual reduction, a 3.6 × 3.7 cm polypoid lesion infiltrating into the serosal layer was found, which protruded into, and completely occluded the lumen. The patient underwent a partial enterectomy lasting 2.5 hours, with an estimated blood loss of 20 mL. She had an uneventful recovery, being discharged 8 days postoperatively without any postoperative complications. The patient was closely followed up for 1.5 years postoperatively, during which time she reported no abdominal discomfort. A follow-up abdominal CT scan also revealed no evidence of recurrence.

Pathologic examination showed a 3.6 × 3.7 cm polypoid lesion in the mucosa was present, invading the serosa layers (Fig. 2C). Microscopically, the lesion showed erosions, significant interstitial edema, mucous degeneration, and vascular hyperplasia. A significant number of inflammatory cells with a predominance of lymphocytes, plasma cells, and eosinophils was seen (Fig. 2D). Immunohistochemical examination revealed that it was positive for CD34 and PDGFRα, focally positive for Ki67, and negative for CD117, DOG-1, SMA, S-100, ALK80, ER, PR, and Desmin. These morphological and immunohistochemical findings correspond to an IFP.

## 3. Discussion

Adult intussusception is clinically rare, accounting for about 5% of all intussusceptions.^[7–9]^ Imaging plays an important role in the diagnosis of intussusception in adults. Barium enema angiography can not only confirm the diagnosis of intussusception, but also reset the intussuscepted intestinal tube, which has both diagnostic and therapeutic roles, but it is not helpful for small bowel-type intussusception. Abdominal ultrasound is fast, noninvasive, simple, and has high specificity for all types of intussusception. The diagnostic accuracy of abdominal ultrasound for intussusception can reach more than 75%, especially for patients with palpable abdominal masses.^[10]^ Abdominal CT is the most important imaging examination for adult intussusception, which can confirm the diagnosis by obtaining specific signs through CT, such as the “target sign” and “cuppy sign.” At the same time, abdominal CT can further understand the cause of intussusception, the location, and the degree of intestinal luminal dilatation, and determine the presence or absence of distant metastasis and surrounding lymph nodes for potentially malignant lesions, which can make up for the shortcomings of abdominal ultrasound and barium enema. For intussusception, aggressive surgical exploration is essential. The 2 patients were diagnosed with intussusception by abdominal CT and underwent partial small bowel resection. The 2 patients were very satisfied with the outcomes of surgical treatment.

IFP can be found throughout the gastrointestinal tract but is more frequent in the stomach (66.7%), followed by the small intestine (21.1%) and colon (8%), and is rare at other locations(<3%).^[2]^ The 2 cases of IFP were both located in the small bowel, 1 in the jejunum and the other in the ileum, which is rare. The etiologies and pathophysiological mechanisms of IFP have not been fully defined. As reported in the literature radiotherapy, local infection (worm, helicobacter pylori), allergic reaction, autoimmune processes, or excessive host response to an unknown stimulus have all been described as possible causes of IFP development.^[2,11,12]^ Some studies suggest IFP is a benign neoplasm driven by activating mutations in the tyrosine kinase receptor PDGFRA, and there are usually oncogenic PDGFRA mutations with a certain degree of familial aggregation.^[13,14]^ Regrettably, we did not perform a gene mutation analysis for the 2 cases.

Morphologically, most IFPs present as a solitary, sessile, or pedunculated polypoid mass emerging from the deep or submucosal layers of the mucosa. For the 2 cases, IFP broke through the submucosa, 1 invading the muscular layer, and another even invading the serosa layers, without peripheral lymph node enlargement. It has been reported that IFP can invade the serosa through the lamina propria, and transmural growth occurs, which is an exceedingly rare observation.^[15,16]^ Reports suggest that IFP has potentially aggressive lesions,^[17]^ sometimes with peripheral lymph node enlargement.^[18]^ We found only 4 other previously reported cases, 2 of which were located in the stomach and 2 in the ileum.^[15]^ Although the spread of IFP under the muscularis propria is extremely rare, the identification of similar cases and further study will enhance our understanding of the nature of this tumor. A study found that among 50 cases of gastric IFPs, 4 instances (8%) were associated with concurrent adenocarcinoma or adenoma in the same anatomical region.^[19]^ A comprehensive review of the literature revealed 3 documented cases of recurrent IFPs within the gastrointestinal tract.^[20–22]^ Although there is currently no direct evidence to suggest that IFPs are malignant neoplasms, the characteristics of invasive growth pattern, recurrence, and association with adenocarcinoma or adenoma reported in the aforementioned literature, strongly support the malignancy nature of IFPs.

IFP typically shows spindle-shaped tumor cells surrounding small blood vessels and mucosal glands, forming a classic onion-like concentric structure, with eosinophil infiltration in the background. Immunohistochemical staining of IFP is invariably positive for vimentin and usually positive for CD34, which is the most useful marker to confirm the diagnosis. However, when an IFP presents without typical morphological features such as “onion skinning” and abundant eosinophils, nor positive CD34, it is difficult to diagnose.^[2,23]^ Besides, an atypical IFP, lacking CD34 expression and “onion skinning,” and harboring an activating PDGFRA mutation has been documented.^[23]^ For the first present case, immunohistochemistry was negative for CD34. However, the results of the consultation revealed that it was weakly positive for CD34, RNA-Seq analysis detected no fusion gene, and proliferative spindle mesenchymal cells were seen with eosinophilic infiltration, which contributed to the diagnosis of an IFP. CD34 expression in the second case is positive typically, which is vital for the diagnosis. If the IFP expresses negative for CD34, the differential diagnosis must be established with other spindle cell lesions of the gastrointestinal tract such as gastrointestinal stromal tumor (GIST), leiomyoma, schwannoma, solitary fibrous tumor and inflammatory myofibroblastic tumor (IMT). GISTs, despite their morphological diversity, rarely exhibit an inflammatory background with lymphocytic, plasmacytic, or eosinophilic infiltration. Immunohistochemically, they are typically positive for CD117 and DOG-1.^[24]^ Leiomyomas occurring in the gastrointestinal tract typically do not exhibit an inflammatory background, and immunohistochemically, they consistently express myogenic markers such as SMA, desmin, and myoglobin, but do not express CD34.^[25]^ Solitary fibrous tumors typically present with alternating areas of cellular sparsity and density, characteristic arborizing blood vessels, and immunohistochemical expression of CD34, bcl-2, and CD99.^[26]^ IMTs typically present with spindle cells arranged in a woven or storiform pattern, with a stroma rich in inflammatory cells. Vascular whorls or an “onion-skin” appearance is rarely seen. Approximately 50% of IMTs express ALK immunohistochemically but do not express CD34.^[27]^ Schwannomas typically have a capsule, with a lymphocytic cuff-like structure visible at the capsule site. Immunohistochemically, the spindle cells consistently express S-100 protein but do not express CD34.^[28]^ Besides, a rare tumor occurring in the gastrointestinal tract with an inflammatory background is follicular dendritic cell sarcoma, which is characterized by the constant expression of CD21, CD23, and CD35.^[29]^

## 4. Conclusion

In conclusion, we described 2 rare cases of small bowel intussusception caused by IFP, which invaded the muscularis propria and the subserosa layer. IFP’s invasive nature is extremely rare, providing additional evidence to support the neoplastic nature of IFP, because these lesions commonly involve only the submucosa. CD34 is the most useful marker to confirm the diagnosis, however, not all IFPs stain positive for CD34. When an IFP is negative for CD34 expression, differential diagnosis becomes crucial.

## Acknowledgments

This work was supported by the National Natural Science Foundation of China (grant no. 82303479) and the Dalian Peak Climbing Plan Construction Project.

## Author contributions

**Data curation:** Yuqiang Tang, Yu Chen, Boyang Dai.

**Funding acquisition:** Xinye Cui, Fuwen Luo.

**Resources:** Yu Chen, Boyang Dai.

**Software:** Yu Chen.

**Writing – original draft:** Yuqiang Tang, Xinye Cui.

**Writing – review & editing:** Zhengdong Zhao, Fuwen Luo.
